# Regulation of nitric oxide generation and consumption

**DOI:** 10.7150/ijbs.105016

**Published:** 2025-01-13

**Authors:** Husam M Abu-Soud, Olivia G Camp, Jayanth Ramadoss, Charalampos Chatzicharalampous, George Kofinas, Jason D Kofinas

**Affiliations:** 1 Departments of Obstetrics and Gynecology, The C.S. Mott Center for Human Growth and Development, Wayne State University School of Medicine, Detroit, Michigan 48201, USA.; 2 Department of Physiology, Wayne State University School of Medicine, Detroit, MI, 48201, USA.; 3 Department of Microbiology, Immunology and Biochemistry, Wayne State University School of Medicine, Detroit, MI, 48201, USA.; 4 Kofinas Fertility Group, 65 Broadway, 14th floor, New York, NY 10006, USA.

**Keywords:** Nitric oxide deficiency, nitric oxide synthase, mammalian peroxidase, NOS inhibitors, ROS

## Abstract

Nitric oxide (NO), originally discovered for its role in cardiovascular function, is a key molecule in physiological processes including metabolism, neurotransmission (including memory, learning, neuroprotection and synaptic plasticity), immunity, reproduction, and much more. NO can be synthesized by the catalytic activity of the enzyme nitric oxide synthase (NOS), which is found biologically in three isoforms, or nonenzymatically based on simple reduction of nitrate and nitrite or by the NO-donor S-nitrosothiol (R-SNO). Importantly, the deficiency of NO has been noted in a wide range of pathologies including cardiovascular disease, cancer, erectile dysfunction, male and female infertility, and mitochondrial disease. While there are several pathways that can lead to a reduction in the bioavailability of NO (i.e., consumption, inhibition, and substrate competition) it is the conclusion of the authors that multiple pathways co-exist in pathological states. This article outlines for the first time the major pathways of NO generation, the importance of NO in health, NO scavenging and enzyme inhibition, and the potential benefits of supplementation.

## 1. Introduction

Nitric oxide (NO) is a signaling molecule and a powerful vasodilator. It plays a critical role in hormonal release stimulation and neurotransmission regulation 1, 2. NO is not only generated enzymatically by nitric oxide synthases (NOSs) [neuronal NOS (nNOS), inducible NOS (iNOS), and endothelial NOS (eNOS)] but also is generated non-enzymatically either by one and two electron reductions of nitrite (NO_2_^-^) and nitrate (NO_3_^-^), respectively or via the NO-donor S-nitrosothiol (R-SNO). The three NOS isoforms can be distinguished from each other by their primary sequence, post-translational modifications, cellular location, and tissue expression 3. For example, eNOS and nNOS are constitutively expressed, whereas iNOS expression is induced. It has been suggested that crosstalk exists between the three isoforms to regulate NO production in both healthy conditions (physiological NO signaling) and in disease states (excessive NO generation). This is accomplished primarily through coordinated expression, subcellular localization, and activity levels 4, 5. While there is ample evidence for crosstalk between the isoforms, a unifying concept of regulation and the factors that govern their concentrations in different biological compartments is lacking. Neuronal NOS is constitutively expressed in the brain and is made up of both particulate and soluble forms that qualifies these cells to handle various advanced responsibilities such as involvement in memory, modulation of learning, and neurogenesis 3. The expression of nNOS can be found in the central nervous and peripheral nervous systems, as well as skeletal and smooth muscle cells, where it regulates synaptic transmission, central regulation of blood pressure, tone of smooth muscle in blood vessels, and vasodilation via peripheral nitrergic nerves 3. Inducible NOS expression can be found in many cell types in response to lipopolysaccharide, cytokines, or other agents and contributes to the pathophysiology of inflammatory diseases and septic shock 3. The primary role of iNOS is in the immune response where NO can break up or inactivate heme-containing enzymes or iron-sulfur clusters, modulate cysteine nitrosylation, or can react with superoxide (O_2_^•-^) to form peroxynitrite (ONOO^-^), a potent oxidant and nitrating agent 6. Both NO and ONOO^-^ can cause DNA breaks and damage by invading pathogens. Moreover, NO can activate the second messenger cyclic guanosine monophosphate (cGMP) through mediation of guanylate cyclase activity that then activates downstream signaling cascades 6. Endothelial NOS is regularly expressed in endothelial cells where it functions mainly to keep blood vessels dilated, regulates blood pressure, displays several vasoprotective functions, and acts as an anti-atherosclerotic agent 3. Endothelial cell eNOS activation is mediated by post-translational modification via its multi-site phosphorylation. For instance, ser1177eNOS is a widely investigated excitatory phosphorylation site in the enzyme's reductase domain, whereas thr495eNOS is an inhibitory site located in its calmodulin binding domain 7. NO generated in endothelial cells diffuses rapidly to adjacent smooth muscle cells, which activates soluble guanylate cyclase (sGC) and allows the conversion of GTP to cyclic GMP, initiating a signaling cascade leading to vasodilation 8, 9.

**Figure 1:**
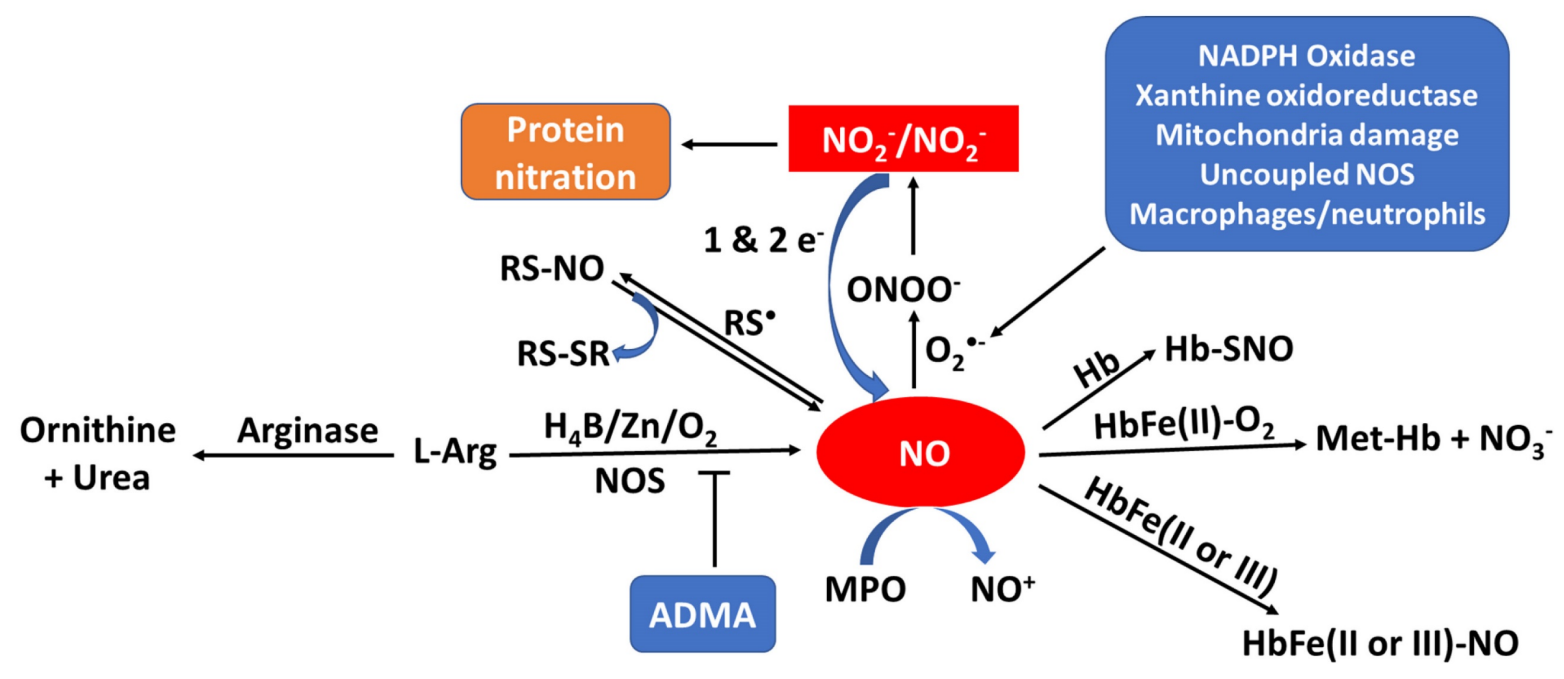
Model outlining the pathways of NO production, consumption, and NOS inhibition and their byproducts. Sources of NO production include NOS, nitrite/nitrate (NO_2_^-^/NO_3_^-^) reduction, and R-SNO. Sources of consumption include superoxide (O_2_^•-^), hemoglobin (Hb), and myeloperoxidase (MPO). NOS inhibition occurs through arginase (enzymatic competition) or asymmetric dimethylarginine (ADMA).

When sufficient NO is not produced, known as NO deficiency, there is an increased risk of several diseases like cancer, diabetes, cardiovascular, pulmonary diseases, mitochondrial diseases, infertility and heart disease 10-17. NO deficiency can often be asymptomatic, while for others the deficiency comes with fatigue and decline in exercise capacity, poor vision, high blood pressure, slow wound healing, weakened immune system, and erectile dysfunction 18-23. An increasing number of disorders are associated with reduced NO synthesis or elevated oxidative removal of NO into the arterial wall, frequently involving those linked with risk factors for atherosclerosis such as high blood pressure, high cholesterol, heart failure, smoking, and diabetes 24. Regardless of whether the underlying etiology of endothelial imbalance is decreased levels of NO generation or enhancement of NO consumption, it has led to the investigation of numerous therapies to assess the reversibility of endothelial dysfunction by NO supplementation and the enhancement of the release of NO from the endothelium. NO supplementation and/or enhancing the release of NO from the endothelium significantly improves cardiac health, healing, erectile dysfunction, high blood pressure, and respiratory response 25. Understanding the mechanisms that cause the deficiency of NO and the effects of low NO levels is essential for addressing this issue and improving overall health. There is considerable evidence, reviewed in this article, to suggest that multiple pathways lead to NO deficiency. As summarized in Figure 1, the bioavailability of NO can be regulated by one or more of the following: NOS uncoupling (deficiency of NOS substrate and/or cofactors), NO scavengers (e.g., oxyhemoglobin, mammalian peroxidases, and superoxide), NOS inhibitors (ADMA), NOS competitor (arginase II), and protein activators (soluble guanylate cyclase (sGC)). In the current review we will discuss the important role of NO in physiological endothelium, and we will highlight the consequences of this molecule in pathological states altering endothelial function 26.

## 2. NO generation

### 2a. Nitric oxide synthases

The enzymatic activity of the three NOSs requires the presence of flavins FMN and FAD, tetrahydrobiopterin (H_4_B), calmodulin (CaM), zinc ion (Zn), NADPH, L-arginine (L-Arg) and molecular oxygen (O_2_) 27 (Table 1a). In the NOS cycle, the electrons donated by NADPH are first delivered to the NOS flavins. In the presence of CaM, electrons transfer instantly to the NOS heme iron 28, 29 where the fully reduced form of NOS permits the heme iron to bind and activate molecular oxygen that catalyzes NO synthesis from L-Arg and H_4_B. L-Arg and the cofactors H_4_B and Zn play a central role in NOS coupling, with their deficiency resulting in the enzyme generating the free radical O_2_^•-^ as a replacement of NO 3 (Table 1a).

### 2b. Nitrite /nitrate

NO can be generated non-enzymatically from the circulating pool of longer-lived NO metabolites including nitrite (NO_2_^-^), nitrate (NO_3_^-^), and S-nitroso and N-nitroso species providing an alternative pathway to supply bioactive NO in addition to typical NOS-derived NO. These metabolites can give rise to NO following reductive bioactivation and cooperates with tissue-bound storage forms of NO undergoing redox-activation to contribute to overall availability 30. One essential contributor of NO_2_^-^ is oral commensal bacteria that possess the ability to bioactivate NO_3_^-^ 30. Thus, the two major sources of NO_3_^-^ supply are NO_3_^-^ from oxidized endogenous NO or dietary intake 31, 32. Nitrite acts as a key regulator of cellular signaling depending on the surrounding oxygen gradient 33. Thus, during hypoxia and low pH situations when NO generation by the NOSs may be compromised, pathways for enzymatic and nonenzymatic reduction of NO_2_^-^ to NO are enhanced 30 by elevation of NO_3_^-^—NO_2_^-^—NO activity. Due to the importance of nitrite in preserving cellular homeostasis, there must be a pathway in situ to maintain its total level in the face of fluctuating levels of dietary intake of nitrite/nitrate and alterations in eNOS activity secondary to fluctuations in blood flow 26. Due to this essential role, dietary NO_2_^-^ is necessary for various physiological activities and having a reservoir of NO_2_^-^ and NO_3_^-^ have positive biological NO-like functions. Thus, dietary NO_2_^-^ plays an essential role in various physiological activities as an effective supplement of NO_2_^-^ and NO in the human body 34.

### 2c. S-nitrosothiol, a NO donor

S-nitrosothiols (R-SNO) are NO donors that extend the lifetime of the active NO by essentially acting as a carrier for the relatively short-lived free radical. SNO is also essential in the localization and activity of enzymes and receptors by encouraging modulation of signaling pathways, axonal transport, synaptic plasticity, and protein assembly 35. The two most commonly occurring and naturally abundant SNO forms are S-Nitroso-serum albumin and S-nitrosoglutathione (GSNO) 35. They are both present in circulating blood and GSNO has also been found intracellularly 35.

S-nitrosolation can occur to any protein with a free thiol moiety but this process is often selective 36, 37. The formation of R-SNO on proteins (namely S-nitrosation or S-nitrosylation) is a unique post-translational modification identified on proteins such as protein tyrosine phosphatases, NF-κB, IκB kinase, Ras, etc. This process is directed by peptide sequences around the cysteine residue, exogenous NO sources, or by compartmentalization of NO synthesis. SNO formation and release is influenced by the redox environment, oxygen and metal ion availability, and thiol reactivity 36. For example, red blood cells release SNO under conditions of low-oxygen 38. There are 3 known mechanisms in which the S-NO bond can be cleaved to release NO. First, copper(I), after Cu^2+^ reduction, can react with the nitrosothiol to form a sulfhydryl anion then regenerating Cu^2+^. Cu^2+^ can be reduced by ascorbate and free thiolate ions, or ascorbate can decompose SNO directly. In this second pathway of NO release, ascorbate concentrations greater than mM level, a free thiol and dehydroascorbate are generated upon NO release. Third, the S-NO bond may undergo homolytic cleavage by light (330-350 or 550-600 nm) to release NO and form disulfide bonds 35, 39. Due to the different mechanisms of reduction and nature of RSNO that can alter the rate of NO liberation, concentration, and duration, the physiologic relevance of SNO is still under consideration.

## 3. NOS in health

NO plays a pivotal role in a wide variety of processes ranging from neurotransmission, vasodilation, and platelet function to immunity, penile erection, and modulation of metabolic function through regulation of vascular tone. This function is often attributed to the role of NO in activation of guanylate cyclase (GC). NO binds to GC-Fe(II) to form an unstable six coordinate ferrous-nitrosyl complex which converts to a five-coordinate GC-Fe(II)-NO complex by breaking the trans axial ligand bond, enabling the enzyme to catalyze the conversion of GTP to cGMP. This function is essential in vasculature homeostasis, where eNOS generated NO aids in tissue blood flow regulation, vascular remodeling, and endothelial protection against platelet aggregation and leukocyte adhesion 40, 41. After synthesis, NO diffuses over 100 microns through tissues and enters red blood cells where it reacts with oxyhemoglobin 42. Interestingly, NO removal by a reaction with Hb and O_2_^•-^ does not result in the complete loss of NO-mediated signaling to vascular smooth muscle cells 43, 44, raising the possibility that alternative mechanisms exist for NO depletion.

A loss in NO production results in endothelial dysfunction and represents an early event in the development of hypertension. A disturbance in NOS activity has been attributed to the pathophysiology of heart failure in which it was reported under expression and/or uncoupled NOS activity which promotes the generation of O_2_•- instead of NO that results in dysfunctional calcium (Ca^2+^) handling, cardiac remodeling, hypertrophy, and thus, the development of heart failure 5, 45-48. Conversely, overexpression of eNOS has shown to be protective in animal models 49. Genetic predisposition to enhance NO signaling has been associated with reduced risk of coronary heart disease and stroke 50. Optimal NO production is required, with a fine balance required for many heme and nonheme proteins via interaction with their metal centers 51, 52. Hence, factors that affect rates of NO production and consumption are of significant interest.

Furthermore, nitrosative stress subsequent iNOS induction has been implicated in heart failure 53, 54. NO is a potent scavenger of several radical intermediates such as alkoxyl radicals and lipid peroxyl 44, 55-58. Similar processes likely occur in atherosclerotic lesions where lipid oxidation products are augmented 59. Consistent with this notion, previous studies 60 demonstrated turnover-dependent consumption of NO by 15-lipoxygenase, an enzyme implicated in atherogenesis 61, 62. NO overproduction has the ability to bind with hemoproteins heme iron at nearly diffusion-controlled rates to form the corresponding nitrosyl complex which inhibits their catalytic activity.

## 4. NO scavenging

There are several pathways that can decrease bioavailable NO. First, hemoglobin possesses the physiological ability to bind NO allowing hemoglobin to modulate NO activity, maintenance, and signaling function. NO can be depleted by myeloperoxidase (MPO), a pre-inflammatory enzyme, by binding to MPO ferric and ferrous heme iron, or it can be utilized as a one electron substrate for MPO Compounds I (Fe(IIV)=O
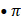
^+^ and Compound II (Fe(IV)=O complex) generating nitrosonium cation (NO^+^) 44, 52. Asymmetric dimethylarginine (ADMA), a recognized NOS endogenous competitive inhibitor, also plays a role in the pathogenesis of lowering NO generation in several disorders 63. ADMA is a competitive inhibitor of NOS binding to the L-Arg site, disturbing the function of the enzyme and resulting in the production of O_2_^•-^ instead of NO. Below, we will discuss in detail the mechanisms in which NO can be depleted.

### 4a. Hemoglobin and NO scavenging

Nearly 92% of red blood cells are made up of hemoglobin (Hb), an oxygen-carrying protein that is responsible for their red color 64. Hb is made up of a tetramer containing four protein globin subunits each with a heme prosthetic group allowing it to bind oxygen in two forms: the taut form (T-form) and the relaxed from (R-form) 65. The T-form has a lower oxygen affinity while the R-form has a higher oxygen affinity, and with the ability to spontaneously change from one form into another, allows Hb to transfer molecular O_2_ from the lung to the outlying tissue, and carbon dioxide from tissue to the lung 66. NO can also bind to the ferrous heme on the cysteine thiol at the beta-93 position of Hb forming the corresponding HbSNO complex (Table 1b). SNO-Hb can also be formed through a reaction of NO with a thiyl radical. Formation of SNO-Hb takes place when the Hb is in the R-state 67-71 and NO released from the complex is linked thermodynamically to the release of O_2_. Notably, the reactivity and function of this Cys is linked to the binding of oxygen (i.e.'thermodynamic linkage').

In its T-state, α-nitrosyl hemoglobin has NO binding to the heme iron which causes histidine and iron to break forming a five-coordinated Hb-Fe(II)-NO complex (one bound to the NO and four bound to the heme porphyrin ring) and this species can be detected directly by electron paramagnetic resonance (EPR) technique 72. NO has a very high affinity for the ferrous heme, with a dissociation constant (K_d_) of 10^-10^-10^-11^ M. NO can also bind to the methemoglobin to generate Hb-Fe(III)-NO complex with a considerably lower affinity (K_d_=2.5 × 10^-4^ M) (Table 1b) 42. The functional Hb that displays high capacity to deplete NO typically exist in two ferrous forms: oxygenated (oxy-Hb; Hb-Fe(II)-O_2_) and deoxygenated (Hb-Fe(II)). Hb ferrous (Fe (II)) displays high oxygen affinity with a carrying size ranging from 1.36 to 1.37 ml O_2_/gram of the protein. Methemoglobin (Fe-(III)), ferryl porphyrin radical cation, Hb-Fe(IV)=O^+ π•^), Compound I and (Hb-Fe(IV)=O), Compound II are all other forms of Hb with higher oxidative states that play a role in developing several undesired pathophysiologic disorders 73.

The encapsulation of hemoglobin in red blood cells has previously been shown to scavenge NO. The near diffusion reaction rate between NO and intraerythrocytic hemoglobin plays an essential role in NO bioavailability and alters homeostatic vascular function. One major pathway is through reaction with oxyhemoglobin (that occurs at a second order rate constant of 6-8 × 10^7^ M^-1^ s^-1^) to produce met-hemoglobin and nitrate 74. Through this reaction, the amount of nitrate made is biologically inactive, suggesting a major role of hemoglobin is to inhibit NO signaling. Despite the high affinity of oxyhemoglobin towards NO, several mechanisms have been documented by which hemoglobin may sustain, control, and even generate NO activity leading to NO maintenance of signaling function. These studies have shown that NO can be converted to several functional species including thiols nitrosation, lipid nitration, nitration, and nitrite generation that have been thought to be essential in signal transduction pathways within many different physiological processes 33, 42, 75-78. Due to its essential role in many biological systems, loss of NO bioavailability leads to disease in several circumstances such as hemolytic anemias 79, older blood transfusions 80, and endothelial dysfunction that occurs in pathological conditions like metabolic syndrome, 81 as well as aging 82, 83. In these circumstances, NO supplementation has additional effects that make NO an attractive therapeutic avenue for certain patients. Despite the significant amount of research done in this field, the argument regarding detailed mechanisms of NO activity protection and how Hb contributes to NO activity remains unanswered.

### 4b. Superoxide NO scavenger

NO is thought to act as a protective agent in endogenous anti-oxidative mechanisms by eliminating the toxic radical, O_2_^•-^, thereby helping in the maintenance of the required level of O_2_^•-^ in normal circumstances. This provides a shielding function against the O_2_^•-^action in many organs including the kidney 84. Mitochondrial damage is considered the main generator of intracellular pathologic levels of O_2_^•-^ through NADPH oxidase, which originates in neutrophils, eosinophils, monocytes, and macrophages 85-88. Xanthine oxidoreductase (XOR) another source of reactive oxygen species, converts hypoxanthine and xanthine to uric acid with immediate production of O_2_^•-^ 88, 89. Alternatively, uncoupled NOS mediated by the deficiency of the substrate or NOS cofactors allows NOS to generate O_2_^•-^ instead of NO 28, 90. In all cases, the majority of generated O_2_^•-^ undergoes a nonenzymatic or superoxide dismutase (SOD)-catalyzed reaction generating hydrogen peroxide (H_2_O_2_). Imbalance between O_2_^•-^ production and metabolism may mediate meiotic arrest and apoptotic cell death through activation of caspase-3 with DNA breaks and damage 91. Similarly, compromised antioxidant machinery e.g., reduced glutathione, could affect optimal chromatin decondensation at fertilization and consequently alter gene expression 92, 93. Similarly, DNA repair mechanisms could be altered as well, contributing further to DNA damage 92, 93.

The reaction of NO with O_2_^•-^ occurs at near diffusion rate yielding ONOO^-^ and accelerates NO depletion. ONOO^-^ is capable to cross the erythrocyte membrane through anion channels 94, 95 and is classified as a strong oxidant, therefore, it reacts instantly with electron-rich groups including sulfhydryls 96, iron-sulfur centers 97, zinc-thiolates clusters 98, and the active site sulfhydryl in tyrosine phosphatases 99. Due to the fact NO can readily diffuse through membranes, NO and superoxide do not have to be generated within the same cell to form ONOO^-^. Several studies have shown that accumulation of O_2_^•-^ in biological systems can occur in NO deficient-environments and can cause variations in organ function 100. It has been shown NOS inhibition elevates vascular O_2_^•-^ generation both in rats and in humans, and such enhanced O_2_^•-^ production was abolished by the use of a O_2_^•-^ scavenger 101-103. Earlier investigations have shown that during NO inhibition there is increased damage of kidney function in hypertensive animals 104-106. Thus, the development of hypertension may in part be due to disproportion of NO and O_2_^•-^ through their physiologic contributions to controlling normal kidney function 107.

The production of ONOO^-^ from NO consumption is a key mechanism contributing to oxidative stress and is implicated in the pathogenesis of various chronic diseases, making further research in this area crucial for understanding its role in cellular damage. ONOO^-^ is responsible for an increase in the NO_2_^-^: NO_3_^-^ ratio 108, 109 (Table 1c). The pathway of the decay of ONOO- depends on two factors: the pH and bioavailability of carbon dioxide (CO_2_). At physiologic conditions, the CO_2_-catalyzed decomposition of ONOO^-^ is significantly faster than the proton-catalyzed decomposition/isomerization to NO_3_^-^ 110. Many of the biological effects ascribed to NO are in reality mediated by ONOO^-^ and subsequent formation of toxic free radical intermediates. In biological systems where CO_2_ levels are relatively high, the reaction rate with ONOO- is quite fast (rate constant of 3-6 × 10^4^ M^-1^s^-1^). ONOO^-^ and CO_2_ lead to the formation of a short-lived intermediate (lifetime, < 3 ms), nitrosoperoxycarbonate adduct (ONOOOCO_2_-), which decomposes in the absence of target molecules to NO_3_- and CO_2_ through rise of some highly oxidizing radical intermediates, ^•^NO_2_^-^ and ^•^CO_3_^-^ 111, 112 (Table 1c). These free radicals are much more toxic and attack many cellular components, enhancing nitrating capabilities compared with ONOO^-^/peroxynitrous acid (ONOOH), reacting with thiols and iron-sulfur centers, as well as initiating lipid peroxidation.

Although it is necessary to remove unwanted ONOO^-^ to neutralize its toxicity in a variety of pathologies such as cardiovascular and infertility disorders, stroke, diabetes, cancer, and neurodegenerative disorders, ONOO^-^ could also play a role as a strong antimicrobial agent, and therefore, targeting the ONOO^-^-detoxifying systems of microbes appears to be a decent strategy for infection control 113, 114. Striking mechanisms are suggested by which enhanced ONOO^-^/NO, or related physiological changes may induce chronic fatigue syndrome, immune dysfunction, memory dysfunction, and multi-organ pain 115. Several scavengers and neutralizers of ONOO^-^ are currently available such as peroxiredoxins, metalloporphyrins, N-acetylcyteine, and dihydrolipoic acid 116. Consequently, it is possible that in NO deficient diseases, there is an overproduction and accumulation of O_2_^•-^ that lead to development of oxidative stress both in tissues and organs 117.

### 4c. Mammalian peroxidase and NO consumption

MPO, a highly expressed hemoprotein in neutrophils, has been shown to modulate the vascular signaling and vasodilatory functions of NO throughout acute inflammation 118. NO functions to alter peroxidase catalytic activity by generating the labile nitrosoniom cation (NO^+^; lifetime 2 ns) which then decays to nitrite/nitrate 44, 119 (Table 1d). Acute endotoxemia and impaired endothelium in a rodent model, has been shown MPO accumulated in and around vascular endothelial cells after leukocyte degranulation in a dependent relaxant response, to which MPO-deficient mice were resistant 120. Altered vascular responsiveness was attributed to catalytic scavenging of NO by MPO. Therefore, MPO can directly modulate vascular inflammatory responses through regulating NO bioavailability. This is because the mammalian peroxidase superfamily (MPO, EPO, and lactoperoxidase) have proven to be a potent scavenger of NO at sites of inflammation and cardiovascular disorders 44, 119. Functional enzymes utilize H_2_O_2_ in the presence of halides (Cl^-^, Br^-^, I^-^) and pseudo (SCN^-^) to form a redox transient intermediate compound I, a ferryl π cation radical (Fe(IV)=O⋅+π) with a total formal heme charge of +5. Under these circumstances, the oxidation of halides and pseudo halide by compound I occurs through a single 2 e- transfer reaction, where the heme of the enzymes is reduced to ferric state and the corresponding hypohalous acid is formed 121, 122. NO depletion is mediated through direct reaction of NO as 1e- physiological substrate and indirectly by radical-radical coupling reactions with peroxidase-produced free radical species. As a substrate, NO accelerates the formation and decay of compound II, the rate-limiting step in the peroxidase cycle, therefore, enhancing the overall rates of catalysis 44 (Table 1d). It also modulates the distribution of peroxidase intermediates, compounds I and II, available during catalysis, hence affecting the substrate selectivity of the enzymes. In contrast, high levels of NO binds ferric and ferrous forms of MPO to form the corresponding stable six-coordinated low spin, MPO-Fe(III)-NO and Fe(II)-NO complex, respectively, rendering them catalytically inactive 52. Studies of the potential mechanism of interaction between the NO^+^ and DNA bases are crucial for the understanding of the progress of genetic modification. Importantly, it has been thought that the resistance of cancer cells and apoptosis is regulated by NO^+^-mediated S-nitrosylation of key enzymes 123.

### 4d. NOS inhibitor (ADMA)

All three isoforms of NOS can be inhibited by ADMA, released from myelin basic proteins and highly expressed in neuronal tissue. ADMA is thought to be involved in the pathogenesis of decreased NO production in several disorders as it is a competitive inhibitor that binds to the L-Arg site of NOSs, disturbing the function of the enzyme resulting in the generation of O_2_^•-^ instead of NO 124 (Table 1e). It is estimated that around 300 μmol of ADMA/day is generated by humans, and plasma concentrations are estimated around 0.5-5 μmol/L 125. The IC_50_ (half-maximal inhibitory concentration) for the three NOSs is dependent on the prevailing arginine concentration, and ADMA effects can be restored by adding excess L-Arg 126, 127. Because of that, ADMA level has been identified as a marker and independent risk factor for development of atherosclerosis, cardiovascular death and all-cause mortality 128-131. Notably, patients with hypertension who underwent Intensive Lifestyle Treatment (ILT) for six months showed improved in ADMA levels and showed that ADMA was dependent on the dietary inflammatory index (DII) content of the diet 132.

ADMA is typically degraded enzymatically through dimethylarginine dimethylaminohydrolase (DDAH), which occurs in two isoforms DDAH1 and DDAH2 131. DDAH1 is highly expressed in brain, suggesting specific function in this area. The presence of nNOS and DDAH1 in brain suggests that ADMA may have specific central nervous system (CNS) activity and be more than an unregulated metabolite 125. DDAH2 is more abundant in the heart, lungs, and placenta 131, 133, 134. An enhancement of plasma ADMA is associated with decreased NO in individuals with hypercholesterolemia, hypertension, polycystic ovary syndrome (PCOS) and atherosclerosis 124, 131, 135. Serum ADMA levels have small variability during the menstrual cycle, with elevated amounts in the follicular phase and lowering levels in the luteal phase 135. Utilizing dehydroepiandrosterone-induced PCOS rat (Sprague Dawley) model and the ovarian granulosa cell line, KGN, Li and colleagues 136 investigated the effect of the ADMA-dimethylarginine dimethylaminohydrolase 1 (DDAH1) pathway on redox status and ovarian apoptosis. These rats developed high levels of ADMA in serum and lower levels of DDAH1 expression in the ovaries. ADMA treatment of the KGN cells exposure to ADMA stimulate ROS accumulation which mediates apoptosis. Overexpression of DDAH1 enhanced cell viability, and reduce oxidative stress, and this effect was reversed in DDAH1 knockdown cells. Quantification of ADMA levels and redox status in serum specimens obtained from women (n=19) with PCOS and healthy individual (n=17) (controls) have shown that women with PCOS had increased serum ADMA levels and decreased glutathione peroxidase (GSH-PX) relative to controls. Collectively, these investigations highlight the involvement of enhanced ADMA levels and redox imbalance in PCOS, suggesting variations in the activity of DDAH that may restrict NO levels by modulating ADMA 137.

### 4e. NOS Competitor (arginase II)

Arginase is considered as a critical regulator of NOS that may contribute to the progression of multiple pathologies, including vascular disease, aging, cystic fibrosis, sickle cell disease 138. Arginase, a crucial enzyme in the urea cycle, catalyzes the conversion of L-Arg to urea and L-ornithine 139. The ability of arginase to compete with NOS for the same substrate, L-Arg, results in the inhibition of the generation of NO through NOS uncoupling (Table 1f). Under conditions of NOS uncoupling, such as in the absence of the substrate L-Arg and/or the cofactors zinc or H_4_B, the ferric resting NOS undergoes steady-state catalysis of NADPH oxidation. The NOS will still undergo heme iron-catalyzed O_2_ reduction but will generate superoxide and/or ONOO^-^ instead of NO and no ferrous-nitrosyl complex was formed during study state catalysis of NOS 138. The competition also causes restraint of the translation and stability of iNOS proteins and inhibition of iNOS activity throughout the urea production life cycle. Arginase also diverts the metabolism of L-Arg to L-ornithine and the generation of polyamines and L-proline which are important for smooth muscle cell growth and collagen synthesis 138. Enhancement of arginase activity inhibits eNOS NO synthesis and may lead to endothelial dysfunction in several disorders including hypertension, diabetes, aging and ischemia-reperfusion, as initiation of arginase may encourage abnormal vessel wall remodeling and neointima formation 140. Growing evidence suggests arginase may stimulate both endothelial and vascular smooth muscle cell dysfunction by altering the intracellular metabolism of L-Arg 138. In circulation, the synthesis of NO by eNOS plays an important role in protecting vascular homeostasis by inhibiting vascular tone, platelet aggregation, as well as inflammation 41. iNOS NO functions in an autocrine manner to limit collagen production and the medial expansion of smooth muscle cells by blocking cell growth and inspiring apoptosis 141. Thus, arginase is characterized as a promising novel therapeutic target in the reversal of endothelial and smooth muscle cell dysfunction and for the prevention of vascular disease.

## 5. NO supplementation

Several studies have shown NO supplementation provides a remarkably enhanced respiratory response, which significantly elevates the speed throughout phase II of pulmonary O_2_ utilization at the start of moderately intense endurance exercise. Other functions include better recovery after major injury or trauma, prevention of the common cold, reduction in the side-effects of memory loss, and the effective healing of diabetic foot ulcers 25. Increasing the intake of vegetables high in NO_3_- such as celery, arugula, beetroot, and spinach, can help boost NO in the body, as well as participating in normal exercise that in turn improves endothelial performance and, therefore, natural NO production 142. NO supplementation through direct NO inhalation (approved by the US Food and Drug Administration (FDA)), or through a L-Arg/citrulline or dietary supplementation for improving NOS or enhancing NO bioavailability may help improve cardiac health, reduce erectile dysfunction, improve ovulation, enhance performance during exercise, reduce high blood pressure during pregnancy, and improve healing processes and the respiratory response 25. For example NO inhalation and NO generated by electrochemical reduction of NO_2_^-^ using copper catalyst (copper (II)-tri(2-pyridylmethyl) amine (Cu(II)TPMA) complex) have been used as a mediator, for insistent pulmonary hypertension of newborn babies (PPHN) 143. This treatment has been shown to improve oxygenation and reduce the requirement for higher-risk extracorporeal membrane oxygenation (ECMO) therapy. Direct inhalation NO not only promotes privileged pulmonary vasodilation and lowers pulmonary vascular resistance, but also has a beneficial effect on treatment of other illnesses including pneumonia, stroke, and acute respiratory distress syndrome (ARDS) 144. Inhalation has recently been used as antiseptic agent in the treatment of cystic fibrosis and tuberculosis, and as an anti-inflammatory agent to modulate immune response and promote survival in patients with malaria 145. Inhalation NO has also been shown to provide neuroprotection and ease brain damage 146. NO supplementation improves lung function in cystic fibrosis patients undergoing treatment for altitude sickness 25.

It is important to note that rare side effects can occur with NO supplementation such as headache, nausea, diarrhea, stomach pain, and heat-palpitations and may cause low blood pressure (hypotension), methemoglobinemia (a rare blood disorder where the blood can't carry oxygen effectively), decreased platelet aggregation (increased bleeding risk), pulmonary inflammation due to the formation of nitrogen dioxide, surfactant dysfunction, and potential for toxicity in sensitive individuals, particularly newborns with certain heart conditions; abrupt discontinuation of NO therapy can also lead to rebound pulmonary hypertension in some cases 25, 147. Supplementation may not be beneficial for those with kidney disease, herpes, and after a person has had a heart attack and it can interfere with certain medications including those for blood pressure. It is recommended to speak with a physician first before starting nitric oxide supplements.

## 6. Conclusion

NO is essential in health as an ideal signaling molecule allowing different cells to communicate and coordinate tissue functions providing the body with the ability to facilitate adaptive changes. Thus, dysregulation in NO production, function, and signaling has been implicated in a variety of pathologies with recent research investigating targeted treatments. While physiological sources of NO include both enzymatic and non-enzymatic pathways, disturbance of either can give rise to an altered REDOX state. Enhanced oxidative stress can negatively affect NOS-NO and cGMP-sGC system through multiple pathways including NOS uncoupling, NO scavenging, and sGC oxidation 40. Understanding the different pathways that cause NO deficiency either by direct NO consumption, or disturbance in NO production by the three NOS isoforms is essential to understanding NO signaling. Importantly, based on the information presented, it is the authors understanding that multiple pathways of NO consumption likely exist simultaneously thus contributing to poorer prognosis in diseases such as cardiovascular disease, in which bioavailable NO is reduced both by the production of MPO and accumulation of free radicals. Therefore, progressing therapeutic options such as through dietary supplements that can overcome NO deficiency such as substrate supplements (i.e., arginine or citrulline), nitrite/nitrate enriched food that may be a good alternative as an arginine precursor, or nutritional adaptations that can support the stability of NO justifies further investigation. Consequently, it is of importance to understand how the imbalances of this crucial molecule can affect a patient's health and clarify conflicting signs and symptoms, with therapeutic treatments supporting medical nutrition.

## Figures and Tables

**Table 1:** Pathways of NO consumption and deficiency. Table outlining the ways in which NO bioavailability can be reduced through enzymatic uncoupling, enzymatic and non-enzymatic scavengers, enzyme inhibition, and enzyme competition with schematic representations of the mechanisms.

NOS uncoupling		
A. Substrate (L-Arg) and co-factors (H_4_B and/or Zn) deficiency	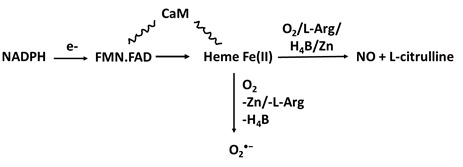	NOS monomarization, Oxidative stress, Protein nitration; DNA damage and biomolecule modification including amino acids, proteins, enzymes, and cofactors; tyrosine nitration.
NO scaenger	Reaction	Effects
B. OxyHemoglobin	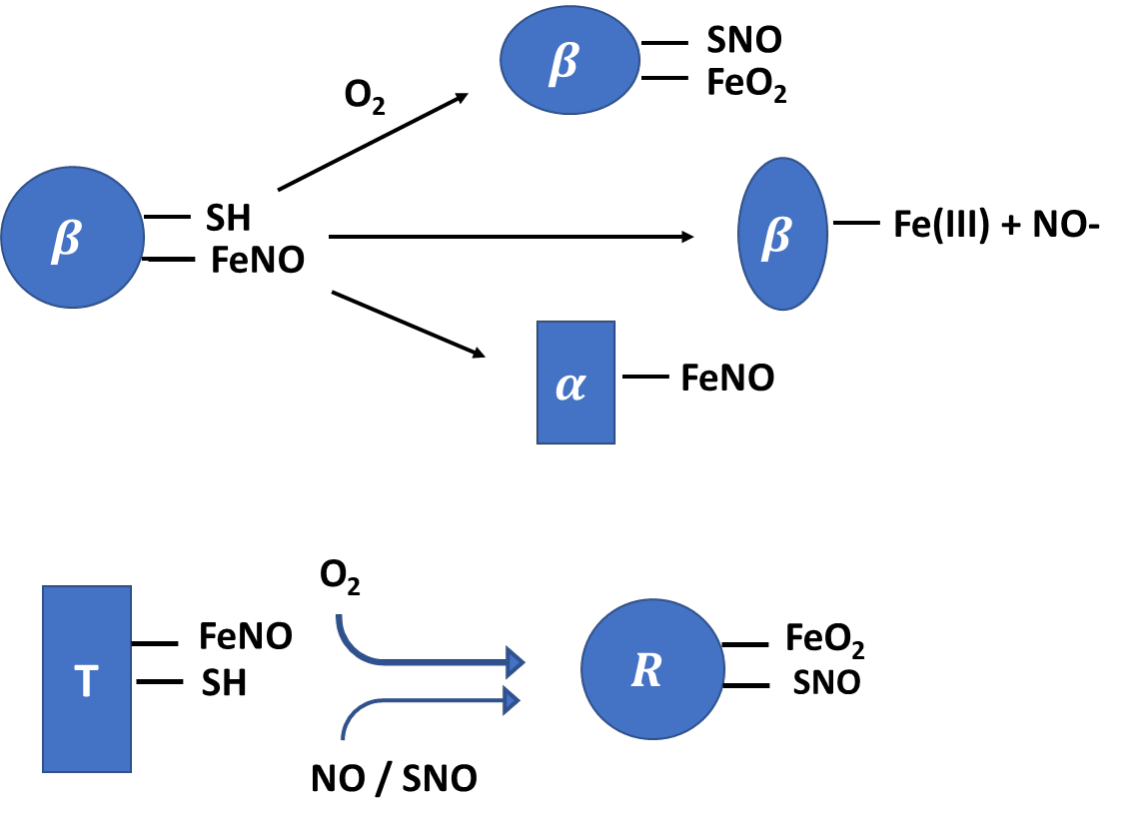	Oxygen deficiency and Hb-SNO formation
C. Superoxide	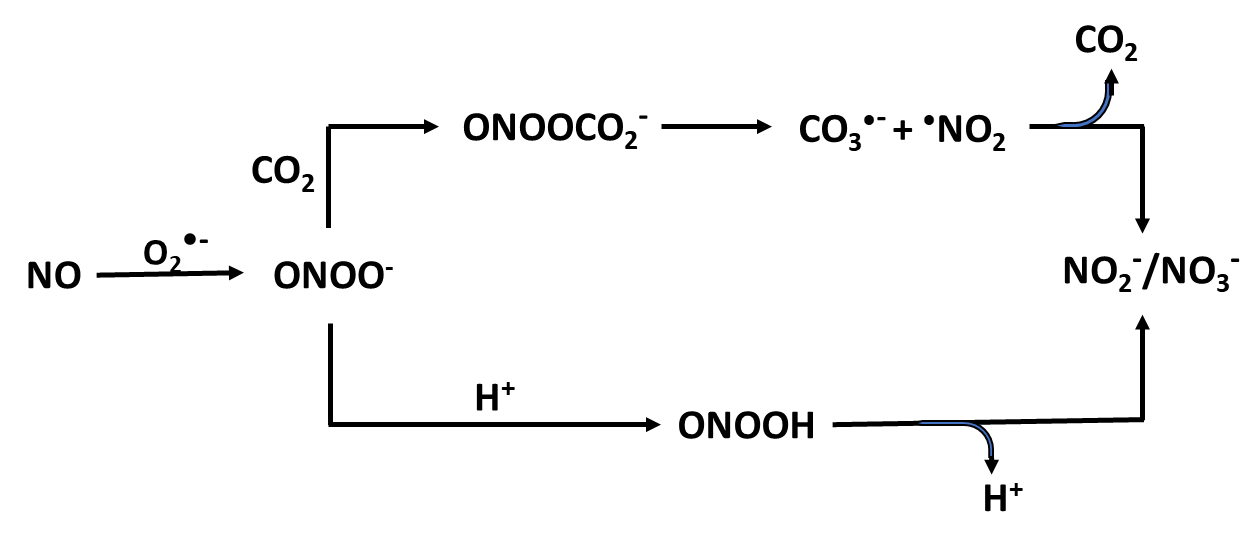	Protein nitration; DNA damage and biomolecule modification including amino acids, proteins; enzyme and cofactor modification; tyrosine nitration
D. MPO	MPOFe(III or II) + NO → MPOFe ((III or II)-NO 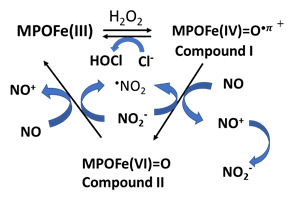	Protein nitrosylation
NOS inhibitors		
E. ADMA	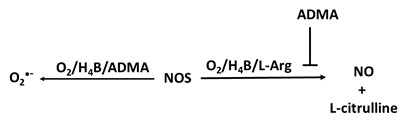	Oxidative stress, DNA damage
NOS Competitor		
F. Arginase II	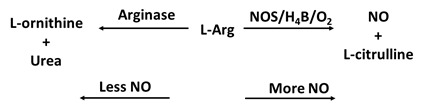	O_2_•- generation

## Abbreviations

NO: Nitric oxide
NOSs: nitric oxide synthases
nNOS: neuronal NOS
iNOS: inducible NOS
eNOS: endothelial NOS
NO_2_^-^: nitrite
NO_3_^-^: nitrate
R-SNO: S-nitrosothiol
ONOO^-^: peroxynitrite
cGMP: cyclic guanosine monophosphate
MPO: myeloperoxidase
ADMA: asymmetric dimethylarginine
O_2_^•-^: superoxide
H_4_B: tetrahydrobiopterin
CaM: calmodulin
L-Arg: l-arginine
Zn: zinc
GC: guanylate cyclase
Hb: hemoglobin
Hb-oxy: oxy-hemoglobin
CO_2_: carbon dioxide
PCOS: polycystic ovary syndrome
